# Clomiphene-induced myocardial infarction in a young male: A case report of a rare cardiovascular complication

**DOI:** 10.1177/00368504251349392

**Published:** 2025-07-09

**Authors:** Zubair Shahid, Gi Eun Kim, Tariq Mousa, Ziad AlSehli, Awad Al-Qahtani

**Affiliations:** 1 Department of General Cardiology, 36977 Hamad Medical Corporation, Doha, Qatar; 2 Department of Internal Medicine, 36977 Hamad Medical Corporation, Doha, Qatar; 3 Department of Interventional Cardiology, 36977 Hamad Medical Corporation, Doha, Qatar

**Keywords:** Clomiphene citrate, male hypogonadism, myocardial infarction, drug-induced thrombosis, thromboembolism

## Abstract

Clomiphene citrate is commonly prescribed to treat male hypogonadism due to its ability to increase endogenous testosterone levels while preserving fertility. Although it is generally considered safe, and rare serious cardiovascular events have been reported. We present the case of a male in his early 30s with no known cardiovascular risk factors who developed an acute inferior-posterior ST-elevation myocardial infarction while taking clomiphene for infertility. Coronary angiography revealed complete occlusion of the distal right coronary artery due to a heavy thrombus burden, with no underlying atherosclerotic disease. The patient was successfully treated with aspiration thrombectomy and conservative medical management. This case underscores a potential, albeit rare, association between clomiphene use and myocardial infarction, warranting caution in its use—even among individuals without traditional cardiovascular risk factors.

## Introduction

Clomiphene citrate, a selective estrogen receptor modulator, is commonly used off-label for the treatment of male hypogonadism.^1^ By acting on the hypothalamus and pituitary, it stimulates the release of luteinizing hormone and follicle-stimulating hormone, leading to an increase in endogenous testosterone levels. Unlike testosterone replacement therapy, clomiphene citrate preserves the hypothalamic-pituitary-gonadal axis, thereby maintaining both endogenous testosterone production and spermatogenesis. This unique advantage makes it the preferred choice for patients seeking to preserve fertility.^2^

While clomiphene is generally considered a safe therapeutic agent, post-marketing surveillance has identified potential cardiovascular adverse events, including arrhythmia, chest pain, hypertension, edema, palpitation, phlebitis, pulmonary embolism, portal vein thrombosis,^3^ shortness of breath, tachycardia, and thrombophlebitis.^4^ Clomiphene citrate is considered a safe drug with several studies reporting no significant side effects.^2^ Here, we present a case of acute ST-elevation myocardial infarction (STEMI) in a young male patient undergoing clomiphene citrate therapy for infertility, highlighting the need for clinical awareness of its potential cardiovascular risks.

## Case presentation

### History

A male in his early 30s, presented to the Emergency Department of Heart Hospital, Doha-Qatar via Ambulance with the chief complaint of chest pain for 1 hour duration. The chest pain started while lifting heavy weights during his routine workout at the gym. It was retrosternal, sudden in onset, crushing in nature, severe in intensity, non-radiating, associated with an episode of vomiting, diaphoresis, and difficulty breathing, but not associated with palpitations, blurry vision, or alteration of the level of consciousness. He had no previous history of chest pain and no history of recent emotional or physical stress. He denied history of smoking, alcohol consumption, or illicit drug use. He did not have any cardiac risk factors and there was no family history of coronary artery disease or other cardiac diseases. He exercised regularly at the gym and denied the use of anabolic steroids or growth hormone supplementation. He had been taking clomiphene citrate (50 mg every other day) only for four months for primary infertility.

### Examination

Young gentleman, well-built, with a BMI of 27 kg/m2, who was in mild respiratory distress. He was afebrile. Heart rate was 85 bpm and blood pressure within normal limits, with oxygen saturation of 71% on room air, requiring NIV with FiO2 of 50%. JVP was not raised, and heart sounds were normal with no added sounds or murmurs. Chest examination revealed bilateral basal crackles. There was no pitting edema. The abdomen was soft, lax, and non-tender with no organomegaly.

### Investigations

**Figure 1.**
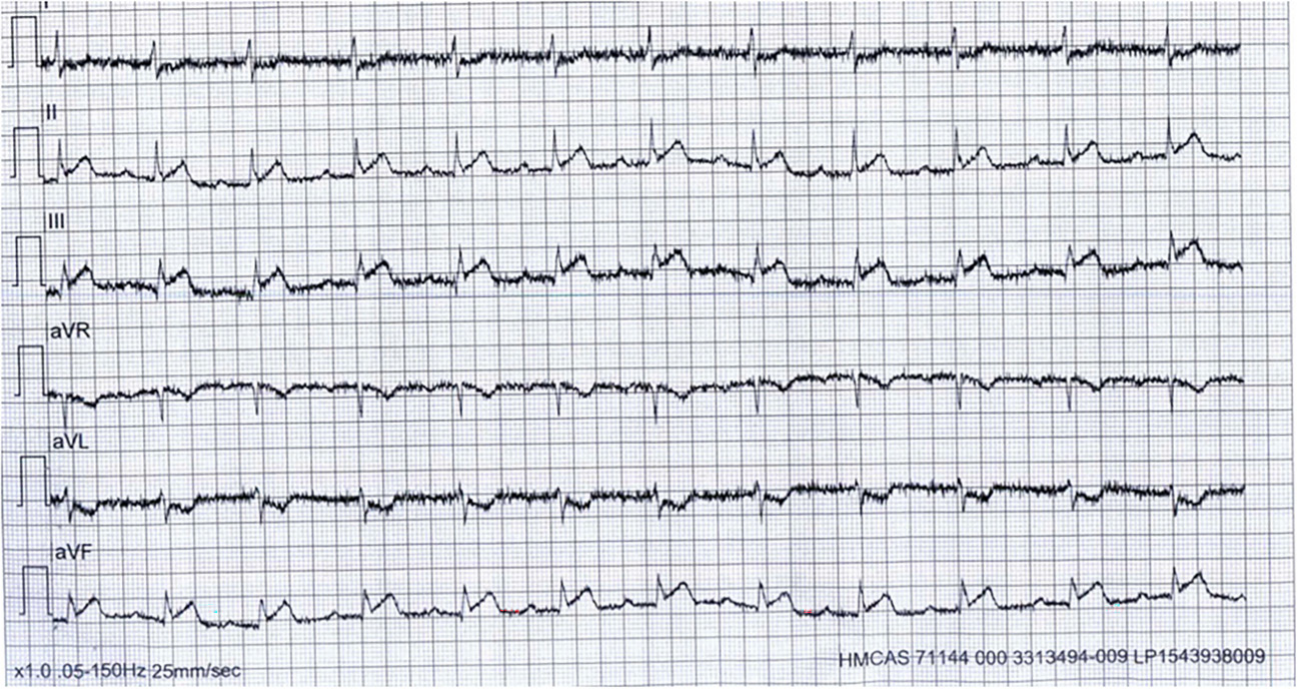
Initial electrocardiogram (ECG) in the ambulance.

**Figure 2.**
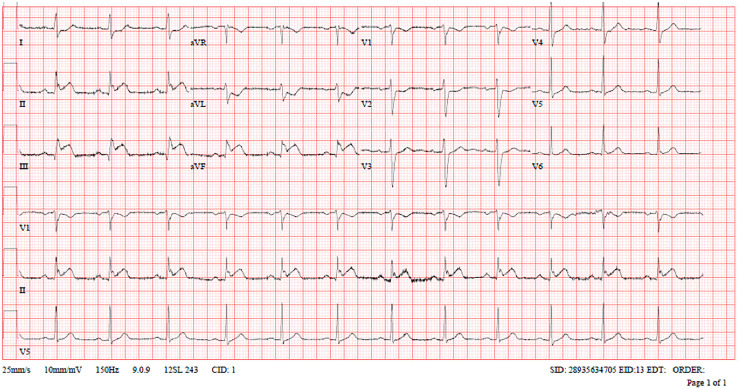
Standard electrocardiogram (ECG) in the Emergency Department.

**Figure 3.**
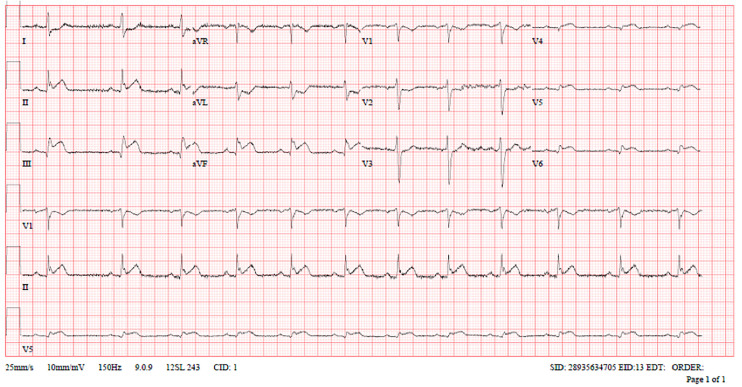
Right-sided electrocardiogram (ECG).

**Figure 4.**
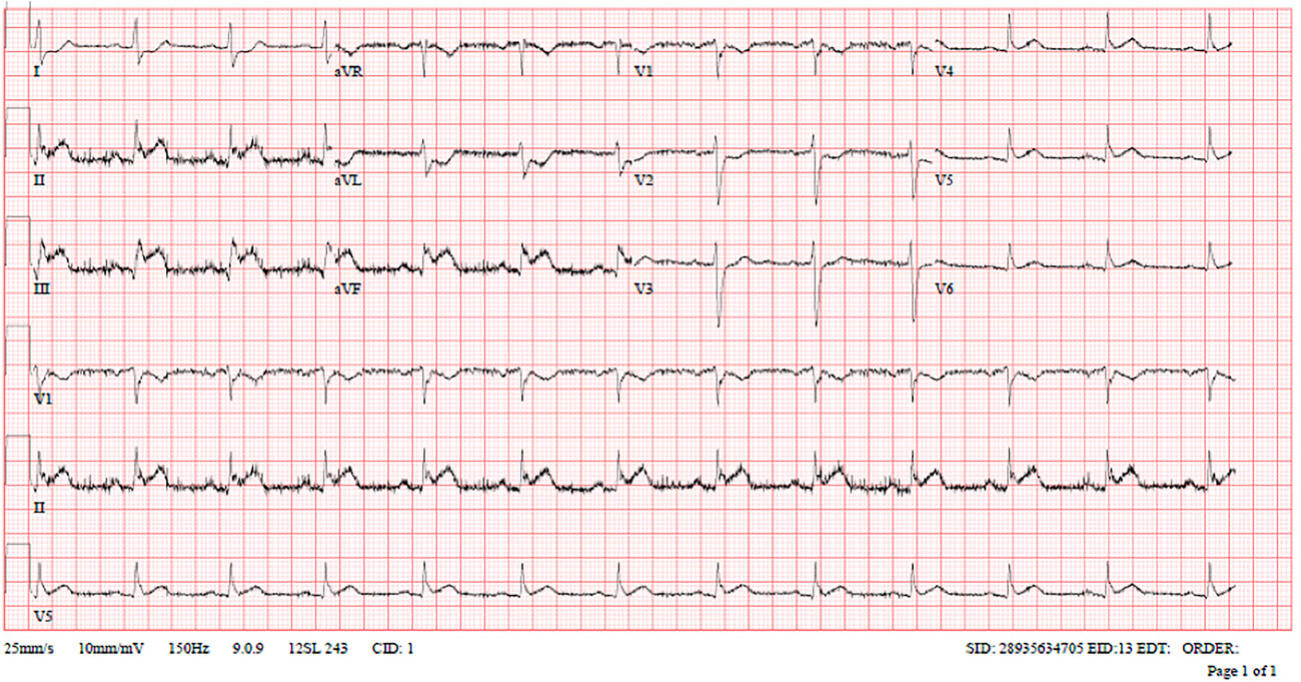
Posterior electrocardiogram (ECG).

- Troponin-T (high sensitivity): 25 ng/L
- LDL-C: 3.3 mmol/L
- HbA1c: 5.3%
- Lactate: 2.7 mmol/L
- ECG: Electrocardiogram (ECG) in the ambulance showed first-degree AV block with ST-elevation in inferior leads and reciprocal ST depressions in Lead I, AVL, and V1-V2 (Figure 1).
- Subsequent ECGs in the Emergency Department showed normal sinus rhythm with ST elevation in inferior, right, and posterior leads with reciprocal changes (Figures 2 to 4).
- Echocardiogram: LVEF 46% with regional wall motion abnormalities in inferior and inferolateral segments.

### Management

**Figure 5.**
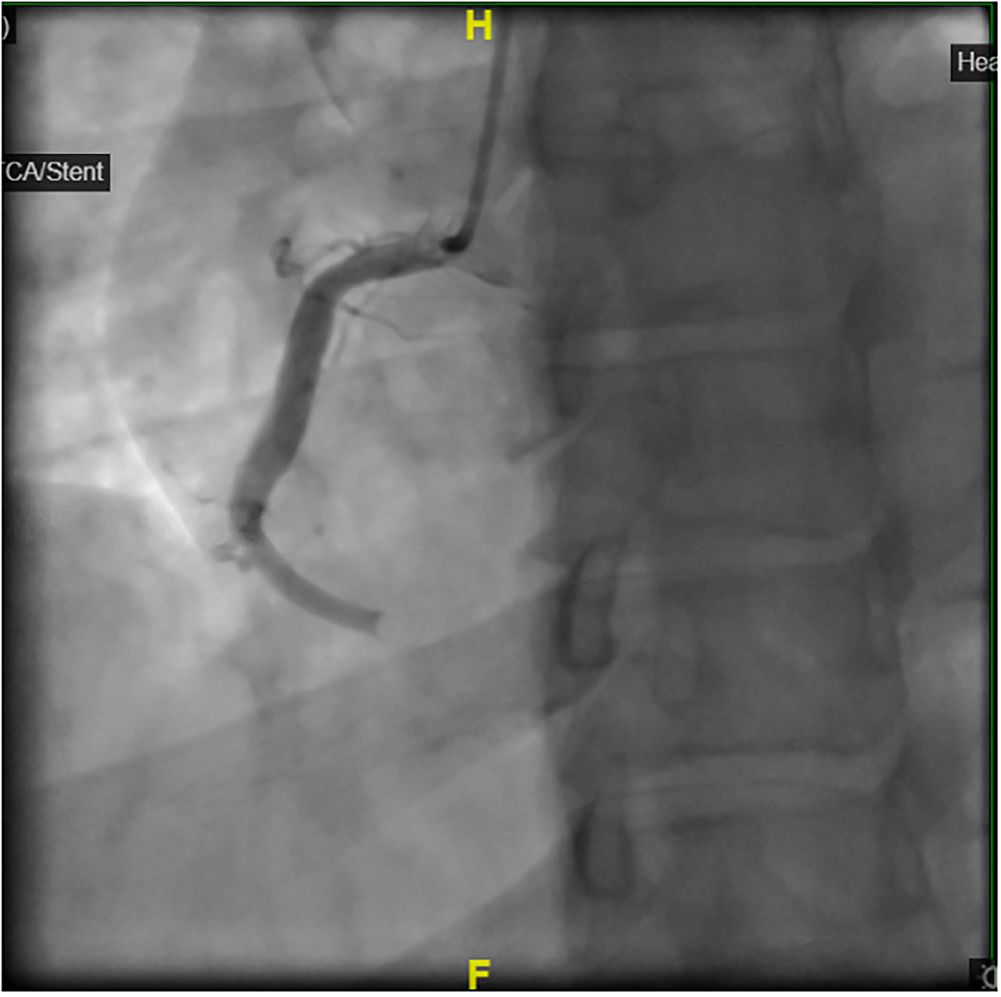
Coronary angiogram showing total occlusion of the distal right coronary artery.

**Figure 6.**
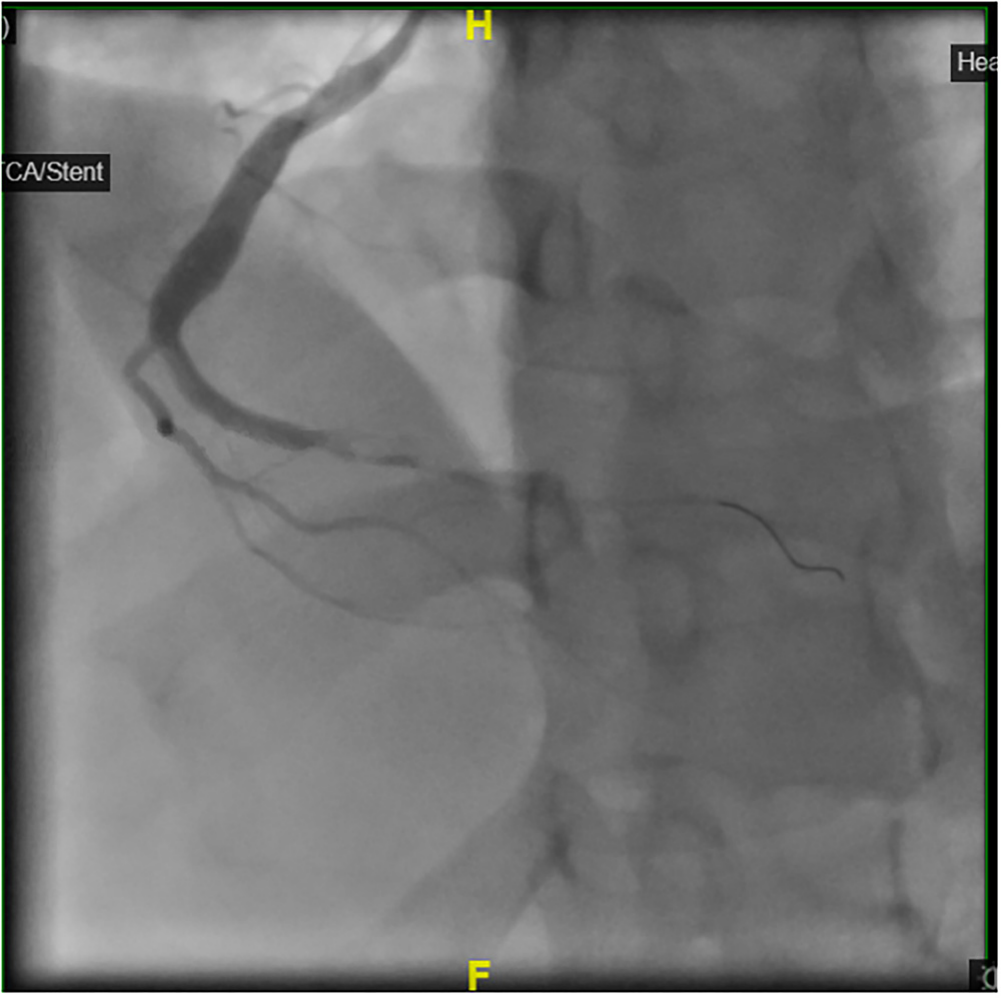
Coronary angiogram showing heavy thrombus burden.

**Figure 7.**
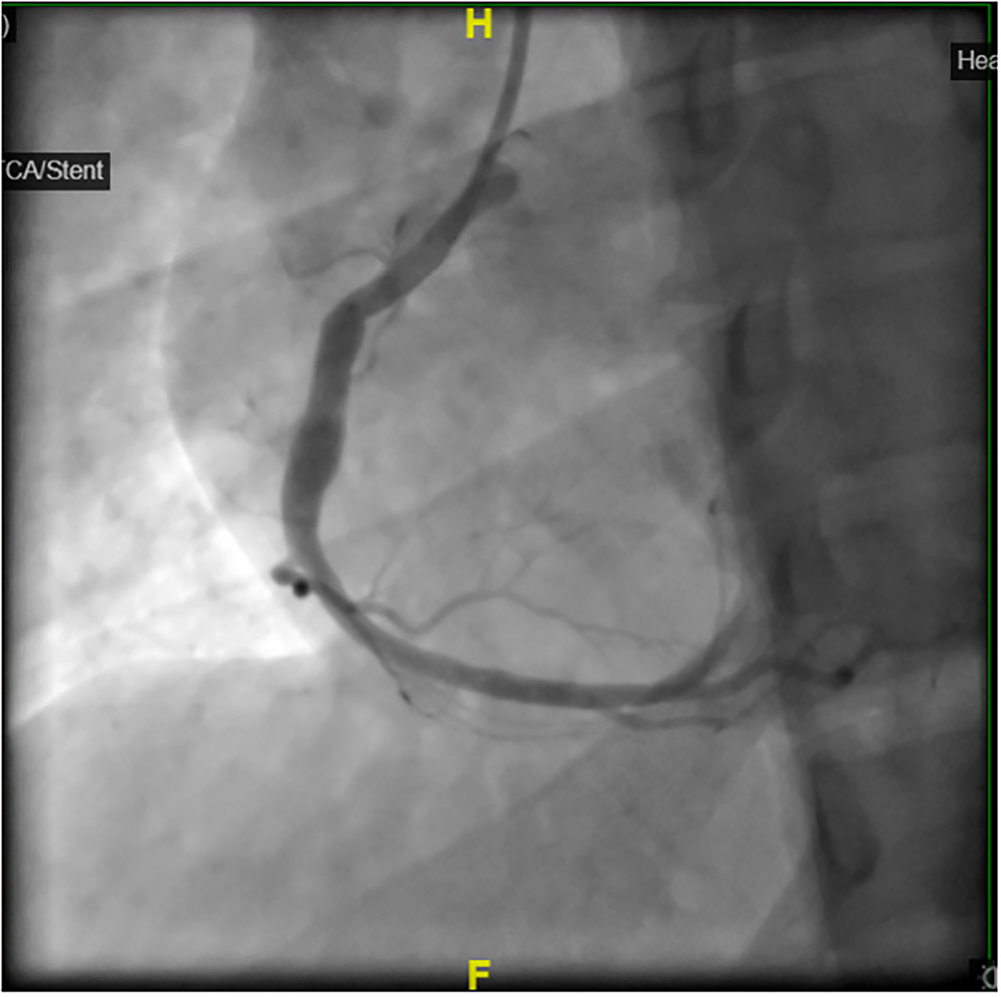
Coronary angiogram post successful PPCI and thrombus aspiration with TIMI 3 flow.

**Figure 8.**
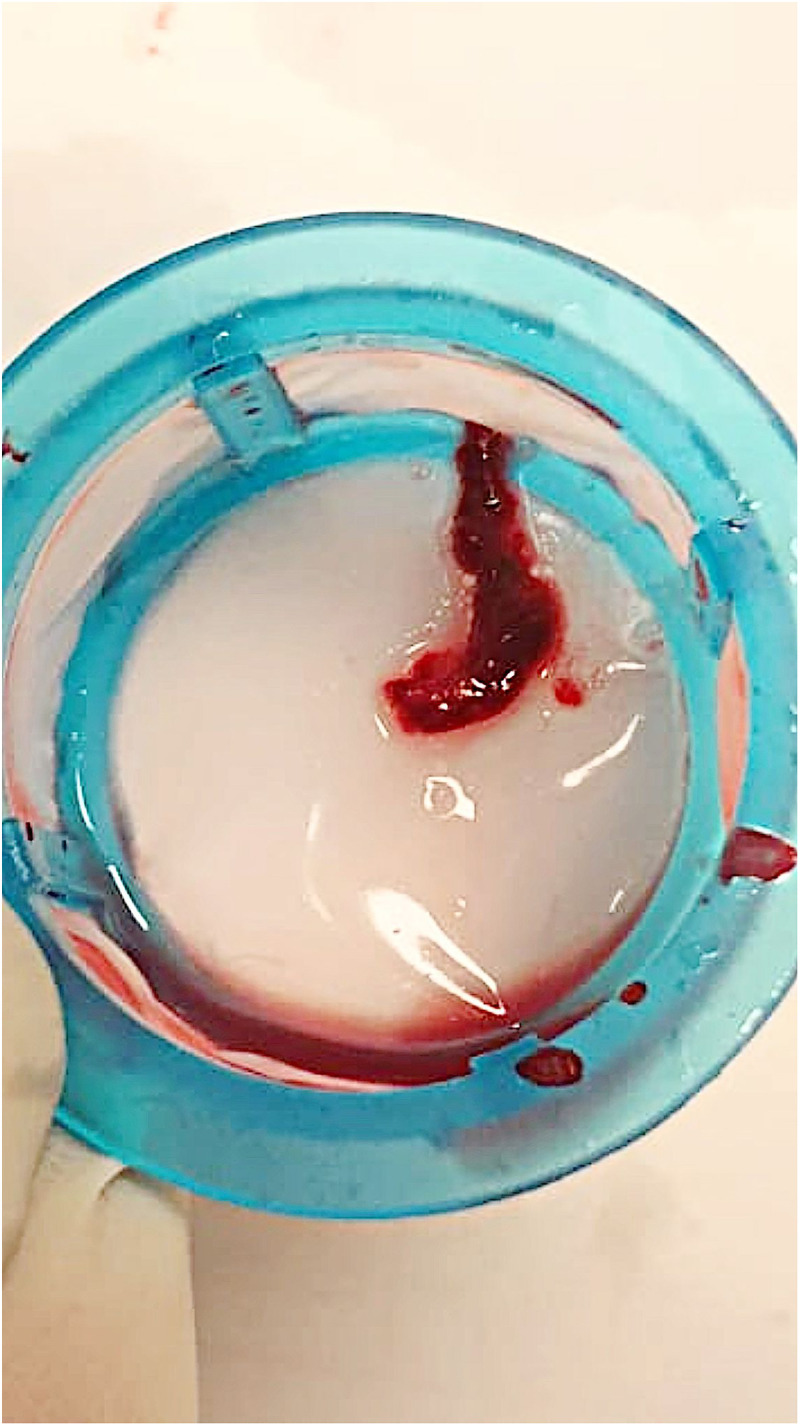
Clots aspirated.

The patient was informed about his diagnosis, consent was taken for the treatment. He has loaded with aspirin 300 mg and clopidogrel 600 mg, IV heparin 5000 units was given and the patient was taken immediately for primary percutaneous coronary intervention (PPCI) where coronary angiogram (CAG) revealed total occlusion of the distal portion of right coronary artery (RCA) with heavy thrombus burden (Figures 5 and 6) and no underlying stenosis. Plain balloon angioplasty (POBA) was performed to distal RCA followed by thrombus aspiration multiple times to extract big clot fragments resulting in good (TIMI 3) flow (Figures 7 and 8). No stent placement was performed due to the absence of a fixed lesion; therefore, medical treatment was recommended and the patient was shifted to the cardiac intensive care unit for overnight observation. Medical treatment with aspirin, clopidogrel, and atorvastatin was initiated. His pulmonary congestion improved with one dose of intravenous furosemide 60 mg with improvement in his oxygen saturation to > 96% on room air. The patient had an uncomplicated recovery with no recurrence of chest pain. Clomiphene was discontinued and the patient was discharged on aspirin, clopidogrel, and atorvastatin. At one-month follow-up, he remained asymptomatic.

PPCI: primary percutaneous coronary intervention; TIMI 3: thrombolysis in myocardial infarction.

The reporting of this case conforms to the CARE guidelines.^5^

## Discussion

Myocardial infarction (MI) in young healthy males with no traditional risk factors for coronary artery disease is uncommon. The incidence of MI in males between the ages of 30 and 34 was 1.29% in one study,^6^ but 90%–97% of young patients with MI had one or more traditional risk factors.^7^ The causes of MI in young patients include atherosclerotic coronary artery disease (CAD), non-atherosclerotic CAD, and recreational drug use.^8^

Plaque rupture accounts for 60%–65% of MI in young individuals and is the most common cause. They are linked to traditional atherosclerotic cardiovascular risk factors, such as older age, family history of coronary artery disease, race, hypercholesterolemia, hypertension, diabetes mellitus, smoking, obesity, and mental stress.^8^ In our patient, none of these cardiovascular risk factors were identified.

Many drugs have been associated with MI, including chemotherapeutics, beta-blockers, aspirin, non-steroidal anti-inflammatory agents, and steroids, as well as recreational drugs such as cocaine, methamphetamine, LSD, heroin, and ecstasy.^9^ However, our patient denied any history of recreational drug use and any prescribed or over-the-counter medications, thus drugs were ruled out as the cause of his MI.

MI can also be caused by spontaneous CAD (SCAD), myocarditis, coronary vasospasm, cardiac embolism, and rarely paradoxical embolism, in patients with normal or near-normal (< 50% obstruction) coronary arteries on the angiogram.^8^ SCAD is a dissection of coronary arteries without atherosclerosis or trauma, and it is most common in young women.^8^ In a coronary angiogram, there was no evidence of dissection, and a culprit lesion with thrombus was identified, ruling out these causes. The echocardiogram did not show signs of patent foramen ovale, and the patient did not complain of any signs of deep vein thrombosis. The patient did not have any evidence of hypercoagulable states, as the hypercoagulability workup was negative*.*

While rare, thromboembolic complications of clomiphene have been documented in both sexes, including cases of deep vein thrombosis, pulmonary embolism, stroke, and retinal vein thrombosis. Only two prior case reports of MI with clomiphene were found, both in female patients.^10,11^ This seems to be the first case report of MI in male patients. The case by Avsar^10^ was similar to our case in that the patient did not have any traditional risk factors for CAD, and the coronary angiogram showed single vessel disease with a heavy thrombus burden, with no significant coronary artery disease in other vessels. The case by Duran et al.^11^ was different in that the patient had a risk of hypercoagulability due to pregnancy, and the coronary angiogram, delayed till the second trimester, was normal, which could be due to the resolution of the thrombus with medical treatment.

Although the exact mechanism of MI in clomiphene use is unclear, clomiphene increases gonadotropin levels and estradiol in men, increasing hypercoagulability.^2^ To support this, there are several reports of thromboembolic complications of clomiphene citrate, including deep venous thromboembolism,^12^ pulmonary embolism,^13^ stroke,^14^ intracranial venous thromboembolism,^15^ and retinal central vein occlusion.^16^ Meanwhile, testosterone replacement therapy in male hypogonadism has not been shown to increase cardiovascular risk.^17^

This case supports a potential association between clomiphene use and coronary thrombosis. The pathophysiology may involve estrogen-mediated prothrombotic changes, although further studies are needed.

## Conclusion

Clomiphene citrate, though generally considered safe, may rarely predispose young men to MI, even in the absence of traditional risk factors. Clinicians should maintain a high index of suspicion when prescribing clomiphene and consider cardiovascular screening in selected patients.
